# Clinical outcomes, molecular epidemiology and resistance mechanisms of multidrug-resistant *Pseudomonas aeruginosa* isolated from bloodstream infections from Qatar

**DOI:** 10.1080/07853890.2021.2012588

**Published:** 2021-12-09

**Authors:** Mazen A. Sid Ahmed, Jemal M. Hamid, Ahmed A. Husain, Hamad Abdel Hadi, Sini Skariah, Ali A. Sultan, Emad Bashir Ibrahim, Abdul Latif Al Khal, Bo Soderquist, Jana Jass, Ali S. Omrani

**Affiliations:** a Department of Pathology and Laboratory Medicine, Division of Microbiology, Hamad Medical Corporation, Doha, Qatar; b The Life Science Centre, School of Science and Technology, Örebro University, Örebro, Sweden; c Department of Medicine, Division of Infectious Diseases, Hamad Medical Corporation, Doha, Qatar; d Communicable Diseases Center, Hamad Medical Corporation, Doha, Qatar; e Department of Microbiology and Immunology, Weill Cornell Medicine-Qatar, Doha, Qatar; f Biomedical Research Centre, Qatar University, Doha, Qatar; g School of Medical Sciences, Faculty of Medicine and Health, Orebro University, Orebro, Sweden

**Keywords:** Bacteraemia, MDR, *Pseudomonas aeruginosa*, Qatar

## Abstract

**Background:**

Bloodstream infections (BSIs) caused by multidrug-resistant (MDR)-*Pseudomonas aeruginosa* are associated with poor clinical outcomes, at least partly due to delayed appropriate antimicrobial therapy. The characteristics of MDR-*P. aeruginosa* bloodstream isolates have not been evaluated in Qatar. Our study aimed to examine *in vitro* susceptibility, clinical and molecular characteristics, and mechanisms of resistance of MDR-*P. aeruginosa* bloodstream isolates from Qatar.

**Materials and methods:**

We included all MDR-*P. aeruginosa* isolated from blood cultures taken between October 2014 and September 2017. Blood cultures were processed using BD BACTEC™ FX automated system. BD Phoenix™ was used for identification, Liofilchem® MIC Test Strips for MIC determination. Whole-genome sequencing was performed using the Illumina-HiSeq-2000.

**Results:**

Out of 362 *P. aeruginosa* bloodstream isolates, 16 (4.4%) were MDR. The median patient age was 55 years (range 43–81) and all patients presented with septic shock. Most patients received meropenem (12/16) and/or colistin (10/16). Clinical response was achieved in eight patients, and five patients died within 30-days. MDR-*P. aeruginosa* isolates belonged to 13 different sequence types. All isolates were non-susceptible to cefepime and ciprofloxacin. The most active agents were colistin (16/16) and aztreonam (10/16). Seven isolates produced *bla*_VIM,_ and four possessed genes encoding extended-spectrum β-lactamases. Aminoglycoside modifying enzymes were present in 15/16, transferable *qnr*-mediated quinolone resistance gene was detected in 3/16, and the novel ciprofloxacin modifying enzyme *CrpP*-encoding gene in one isolate.

**Conclusion:**

MDR-*P. aeruginosa* BSIs are relatively uncommon in Qatar but are highly resistant, harbour multiple resistance genes, and are commonly associated with unfavourable clinical outcomes. Colistin was the only agent with consistent activity against the study isolates.

**Key messages**
- MDR-*P. aeruginosa* constituted <5% of *P. aeruginosa* blood isolates over three years.
- Typical risk factors for MDR infections were highly prevalent in the study population and overall clinical outcomes are consistent with those previously reported.
- Colistin was the only agent with consistent antibacterial activity against the study isolates.

## Introduction

*Pseudomonas aeruginosa* possess a remarkable array of intrinsic and acquired antimicrobial resistance mechanisms, often expressed simultaneously and resulting in multidrug-resistant (MDR) phenotypes [1]. Risk factors for MDR-*P. aeruginosa* infection includes prior antimicrobial therapy, the presence of indwelling medical devices, neutropenia, mechanical ventilation, and previous gut colonization with MDR-*P. aeruginosa* [2]. Bloodstream infections (BSIs) caused by MDR-*P. aeruginosa* are associated with poor clinical outcomes, including prolonged hospitalization, increased healthcare costs, and high mortality [3]. Such outcomes are at least in part due to delayed appropriate antimicrobial therapy and the limited availability of effective treatment options [4]. However, the clinical, microbiological, and molecular characteristics of bloodstream MDR-*P. aeruginosa* isolates have not been evaluated in Qatar. Our aim was to investigate the clinical characteristics and outcomes of MDR-*P. aeruginosa* BSIs from Qatar, assess their *in-vitro* susceptibility, and to investigate their molecular epidemiology and resistance mechanisms.

## Materials and methods

Patients were identified prospectively from routine clinical specimens received by the Division of Microbiology at Hamad Medical Corporation (HMC) in Doha, Qatar. The facility provides routine and tertiary diagnostic services for all primary health centres and public hospitals across the whole country. MDR-*P. aeruginosa* were defined as isolates with *in-vitro* resistance to ≥1 agent from ≥3 antipseudomonal classes of antimicrobials [5]. Consecutive patients with blood cultures yielding growth of MDR-*P. aeruginosa* during the period from October 2014 to September 2017 were included. Clinical data were retrieved from the electronic healthcare system.

Blood cultures were processed using BD BACTEC™ FX automated system (Becton Dickinson, USA). Bacterial identification and initial antimicrobial susceptibility testing were performed on BD Phoenix™ (Becton, Dickinson and Company, Franklin Lakes, New Jersey, United States). Liofilchem^®^ MIC gradient strips (Liofilchem, Roseto degli Abruzzi, Italy) were used for minimum inhibitory concentration (MIC) determination. Broth microdilution was used for colistin susceptibility testing (ComASP Colistin, Liofilchem, Roseto degli Abruzzi, Italy). *Escherichia coli* ATCC 25922, *E. coli* ATCC 35218 and *P. aeruginosa* ATCC 27853 were used as controls. Clinical Laboratory Standards Institute (CLSI) breakpoints were used to interpret susceptibility results [6]. Intermediate and resistant categories were grouped as non-susceptible for all reported antimicrobial agents.

## Whole-genome sequencing and statistical analyses

DNA was sequenced at GATC Service (Eurofins Genomics, Germany) using Illumina HiSeq 2000 system (Illumina, San Diego, California, USA). The genomes were assembled using SPAdes, Version 3.13.0 [7]. Multilocus sequence typing (MLST) of *P. aeruginosa* isolates was performed on MLST server 1.8 provided [8]. The Comprehensive Antibiotic Resistance Database (CARD), Version 1.2.0 were used to annotate antibiotics resistance genes (ARGs) [9]. The pan-genome tree and the k-mer tree were constructed using NDtree [10,11], while Interactive Tree Of Life was used to display, manipulate, annotate, and visualize the phylogenetic trees [12]. PATRIC RASTtk-enabled Genome Annotation Service was used for the detection and annotation of exotoxin genes [13].

Data were presented as frequency or median and range, as appropriate. Statistical analyses were conducted using IBM SPSS Statistics for Windows, Version 25.0 (IBM Corp, Armonk, NY, USA).

## Results

Over the study period, MDR-*P. aeruginosa* constituted 16 (4.4%) out of 362 episodes of *P. aeruginosa* bacteraemia. The included isolates were from blood cultures from Hamad General Hospital (12/16), Rumailah Hospital (3/16), and the National Centre for Cancer Care and Research (1/16). No MDR-*P. aeruginosa* were isolated from blood cultures from other HMC facilities during the study period.

The median patient age was 55 years (range 43–81) and the majority were males 93.8% (15/16). Nine patients were in an intensive care unit (ICU) at the time of MDR-*P. aeruginosa* bacteraemia (Table 1). All patients presented with septic shock and the majority had multiple risk factors for MDR infections, including hospitalization or outpatient hospital attendance within the previous 90 days (15/16), invasive medical devices (13/16), and recent systemic antimicrobial therapy (12/16). The most frequent underlying co-morbidities were diabetes mellitus (11/16), and malignant disease (5/16). Combinations of two or three different antimicrobial agents were used in (10/16) patients. The most commonly used agents were meropenem (12/16), and colistin (10/16). Clinical response was achieved in (8/16) patients, while five patients died of any cause within 30 days of their MDR-*P. aeruginosa* bacteraemia (Table 2).

**Table 1.** Demographics and susceptibility results for patients with MDR-*P. aeruginosa* bacteraemia.

Isolate	Isolation month	Age group	Gender	Hospital	Location	CIP*	ATM*	FEP*	MEM*	CAZ*	TZP*	GEN*	TOB*	AMK*	C/T*	CZA*	CST*
PA84	Feb-15	40–49	Male	HGH	ICU	32	8	32	32	24	48	256	256	32	12	3	1
PA123	Apr-15	40–49	Male	HGH	Inpatient	32	8	32	0.75	12	256	48	64	96	1.5	2	2
PA148	May-15	40–49	Male	HGH	Inpatient	32	8	24	32	6	32	256	32	16	1.5	6	1
PA183	Jul-15	40–49	Male	NCCCR	Inpatient	32	6	16	32	12	256	48	24	32	24	12	2
PA208	Sep-15	40–49	Male	HGH	ICU	32	256	256	32	256	256	256	24	256	256	256	2
PA212	Oct-15	40–49	Male	HGH	ICU	32	256	256	32	256	256	256	32	256	256	256	2
PA220	Oct-15	50–59	Male	HGH	ICU	32	256	256	32	256	256	256	256	256	256	256	1
PA232	Nov-15	50–59	Male	HGH	ICU	1.5	8	256	32	32	256	4	0.75	8	0.75	2	0.5
PA241	Dec-15	50–59	Male	HGH	ICU	3	12	192	32	6	16	6	1	12	1	6	2
PA263	Jan-16	50–59	Male	RH	Inpatient	8	8	32	32	12	64	1.5	0.75	4	1	6	1
PA420	Dec-16	50–59	Male	RH	Inpatient	1.5	24	256	32	96	256	0.75	0.5	3	4	16	1
PA447	Feb-17	60–69	Male	RH	Inpatient	3	16	256	32	48	96	0.75	0.3	2	1.5	8	2
PA457	Mar-17	70–79	Male	HGH	Inpatient	32	16	32	1.5	8	16	1.5	0.75	3	1.5	8	2
PA498	Jun-17	70–79	Male	HGH	ICU	4	1.5	64	32	16	192	256	64	32	256	16	1
PA508	Jul-17	80–89	Male	HGH	ICU	8	3	32	32	48	48	256	256	32	12	24	1
PA527	Aug-17	80–89	Female	HGH	ICU	32	6	256	32	256	256	256	128	256	256	48	1

AMK: amikacin; ATM: aztreonam; CAZ: ceftazidime; CIP: ciprofloxacin; CST: colistin; C/T: ceftolozane/tazobactam; CZA: ceftazidime/avibactam; FEP: cefepime; GEN: gentamicin; HGH: Hamad General Hospital; ICU: intensive care unit; MEM: meropenem; MIC: minimum inhibitory concentration; NCCCR: National Centre for Cancer Care and Research; NS: non-susceptible; RH: Rumailah Hospital; S: susceptible; ST: sequence type; TOB: tobramycin; TZP: piperacillin/tazobactam.

*Minimum inhibitory concentration (MIC) in µg/ml, shading indicates *P. aeruginosa* non-susceptibility to the corresponding antimicrobial agent. Clinical Laboratory Standards Institute (CLSI) breakpoints for susceptibility: CIP ≤1, ATM ≤8, FEP ≤8, MEM ≤2, CAZ ≤8, TZP ≤16, GEN ≤4, TOB ≤4, AMK ≤16, C/*T* ≤ 4, CZA ≤8 and CST ≤2 µg/ml.

**Table 2.** Clinical diagnosis, common associated underlying conditions, and outcome of patients with MDR-*P. aeruginosa* infections from 4 hospitals in Qatar.

Characteristics	Frequency (*N* = 16)
Septic shock	16
Hospital acquired infection	16
Antimicrobial therapy	
Amikacin	1
Aztreonam	1
Meropenem	12
Piperacillin/tazobactam	3
Colistin	10
Number of antimicrobial agents used	
One	6
Two	9
Three	1
Risk factors for MDR-P. aeruginosa infection	
Extensive health care contact^a^	15
Invasive device^b^	13
History of antimicrobial exposure within the preceding 90 days^c^	12
Isolation of prior susceptible *P. aeruginosa*	9
History of MDR infection or colonization within prior 90 days	9
Co-infection with other microorganisms^d^	3
Co-existing medical conditions	
Diabetes mellitus	11
Malignancy	5
End-stage kidney disease	3
Chronic obstructive pulmonary disease	3
Chronic lung disease	2
Organ transplantation	1
Chronic liver disease	1
Outcomes	
Cured	8
Relapsed	2
Died	6
30-Day all-cause mortality	5
90-Day all-cause mortality	1

^a^Extensive health care contact included regular visits to outpatient medical facilities, regular home visit by home care teams, hospitalization within the preceding 90 days, or residency in a long-term care facility.

^b^Invasive devices included central venous lines, ureteral stent, urinary catheter, surgical drain, endotracheal tube, nephrostomy, nasogastric tube, peritoneal dialysis catheter, or gastrostomy tube.

^c^Amoxicillin, azithromycin, ceftriaxone, cefuroxime, ciprofloxacin, clindamycin, colistin, doxycycline, ertapenem, levofloxacin, linezolid, metronidazole, co-trimoxazole, teicoplanin, tigecycline, or vancomycin.

^d^Co-infections were involved the following organisms: *Candida glabrata*, *Klebsiella pneumoniae* (extended-spectrum beta-lactamases), or *Streptococcus agalactiae*.

**Figure 1.**
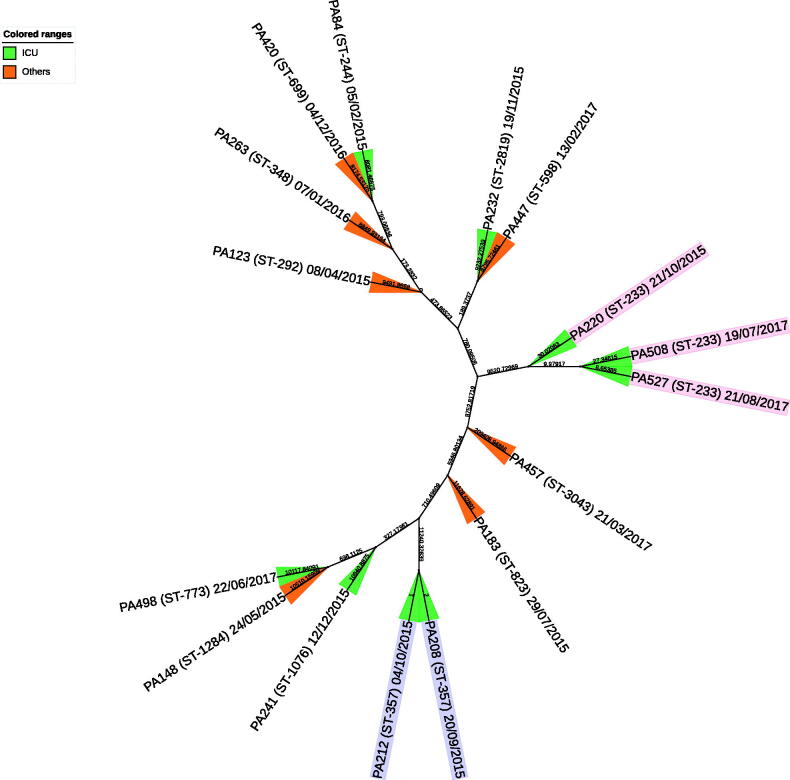
Phylogenetic relationship constructed by comparing the core genome of the16 MDR- *P. aeruginosa* isolates collected from blood stream infection in Qatar between October 2014 and September 2017.

The study isolates belonged to 13 different sequence types. The most frequent were ST233 (3/16), and ST357 (2/16) (Figure 1). We detected *P. aeruginosa*-encoded type III secretion system exotoxins ExoS (9/16), ExoT (15/16), ExoU (6/16), and ExoY (15/16). MDR-*P. aeruginosa* from all patients who died within 90 days possessed three exotoxin-encoding genes (Table 3).

**Table 3.** The main *P. aeruginosa*-encoded exotoxin of the type III secretion system detected in 16 MDR-*P. aeruginosa* isolates from Qatar.

Isolate number	Gene presence (% identity of protein sequences)			
Exotoxin	exoS	exoT	exoU	exoY
PA84*	Yes (100)	Yes (99)	–	Yes (98)
PA123	Yes (100)	Yes (98)	–	Yes (99)
PA148	–	Yes (99)	Yes (99)	Yes (98)
PA183*	–	Yes (99)	Yes (100)	Yes (98)
PA208	–	Yes (100)	Yes (99)	Yes (98)
PA212	–	Yes (100)	Yes (99)	Yes (98)
PA220	Yes (99)	Yes (99)	–	Yes (98)
PA232*	Yes (99)	Yes (99)	–	Yes (99)
PA241	–	Yes (99)	Yes (99)	Yes (98)
PA263	Yes (100)	Yes (99)	–	Yes (100)
PA420*	Yes (100)	Yes (99)	–	Yes (100)
PA447	Yes (99)	Yes (99)	–	Yes (98)
PA457	–	–	–	–
PA498	–	Yes (100)	Yes (99)	Yes (98)
PA508	Yes (99)	Yes (99)	–	Yes (99)
PA527*	Yes (99)	Yes (99)	–	Yes (99)

*Patients with these isolates died within 90 days of MDR-*P. aeruginosa* bacteraemia.

**Figure 2.**
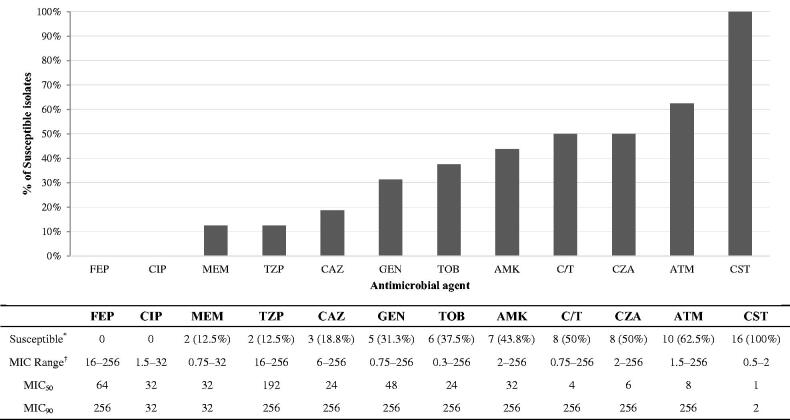
Susceptibility patterns of 16 MDR-*P. aeruginosa* bacteraemia isolates collected from Qatar between October 2014 and September 2017. *Number susceptible (%); ^†^all MIC values are in µg/ml. AMK: amikacin; ATM: aztreonam; CAZ: ceftazidime; CIP: ciprofloxacin; CST: colistin; C/T: ceftolozane/tazobactam; CZA: ceftazidime/avibactam; FEP: cefepime; GEN: gentamicin; MEM: meropenem; MIC: minimum inhibitory concentration; TOB: tobramycin; TZP: piperacillin/tazobactam.

Rates of nonsusceptibility were high to cefepime (16/16), ciprofloxacin (16/16), meropenem (14/16), ceftazidime (13/16), and piperacillin/tazobactam (14/16). The highest susceptibility rates were to colistin (16/16) and aztreonam (10/16) (Figure 2).

Each isolate possessed 1–7 different β-lactamase genes from all classes, including at least one *Pseudomonas*-derived cephalosporinase (PDC)-encoding gene. *bla*_OXA-50_ was present in the majority (12/16), *bla*_VIM_ metallo-β-lactamase (MBL) in seven and extended-spectrum β-lactamases (ESBL) in four isolates (Table 4). All but one isolate possessed up to six different genes encoding aminoglycoside modifying enzymes (AMEs). Most common were aminoglycoside phosphotransferases (APH) (15/16) and aminoglycoside nucleotidyltransferases (ANT) (5/16) (Table 4). Only one isolate carried a gene encoding 16S rRNA methyltransferase (*Rmt*) (Table 5). Fluoroquinolone resistance through topoisomerase mutations *gyrA* (T83I) predominated (13/16), in addition to mutant genes encoding *gyrA* (S80I) and *parC* (S83I) in two isolates, and *parE* (A473V) in one isolate, as well as transferable *qnr* genes in three isolates (Table 5). A mutated *CrpP* (R4K, D7G) gene, which encodes a ciprofloxacin modifying enzyme, was detected in one isolate (Tables 4 and 5). β-lactam resistance through mutated *PBP3* (D350N, S357N) and *OmpK37* (M70I, M128I) were detected in two isolates (Table 5). The active efflux pumps MexAB-OprM, MexCD-OprJ and MexEF-OprN were present in all the study isolates (Table S1, data supplement file).

**Table 4.** Antimicrobial resistance genes detected in sixteen MDR-*P. aeruginosa* bacteraemia isolated between October 2014 and September 2017.

Isolates	PA84	PA123	PA148	PA183	PA208	PA212	PA220	PA232	PA241	PA263	PA420	PA447	PA457	PA498	PA508	PA527
β-Lactamases	Shaded cells indicate presence of gene in the isolates (% of identity of protein sequences)															
CARB-3		99.67														
CTX-M-15					100				100							
SHV-11					100											
VEB-9					99.7	99.7										
VIM-2				100			100							100	100	100
VIM-5					100	100										
PDC-1	100										100					
PDC-3			99.5		99.2	99.24	100					100			100	100
PDC-5		99.75						100		100						
PDC-7				99					99.5					99.5		
OXA-4							100								100	100
OXA-10	100				99.6	99.62										
OXA-50	98.85	98.09			99.2	99.24	99.24	98.85	98.85	99.24	100	99.24	84.73	99.24		
OXA-114a							98.91								100	100
OXA-486																
OXA-488			100													
Aminoglycoside modifying enzymes																
* AAC(6′)-Ib*					100											
* AAC(6′)-II*															100	100
* aadA*	99.62		99.21											99.61		
* aadA2*					99.6		100		100						100	100
* aadA3*		98.48														
* ANT(2′')-Ia*	100		100		100	100								100		
* ANT(3′')-IIa*					99.6	99.63										
* ANT(4′)-IIb*	99.2															
* APH(3′)-IIb*	100		98.51	98.5	99.3	99.25	98.88	98.88	99.25	98.88	99.25	98.51		100	98.88	98.88
* APH(3′')-Ib*	98.88	99.25	99.63	99.6												
* APH(3′)-VIa*					96.1	96.14										
* APH(6)-Id*	99.64		99.28	99.6												
Ciprofloxacin modifying enzyme																
CrpP			96.92													

AAC: aminoglycoside acetyltransferase; ANT: aminoglycoside nucleotidyltransferase; APH: aminoglycoside phosphotransferases; cat: chloramphenicol acetyltransferase; CrpP: ciprofloxacin resistance protein phosphotransferase.

**Table 5.** The resistance genes encoding of target modification in the sixteen MDR-*P. aeruginosa* bacteraemia from Qatar.

Resistance mechanism	Affected antimicrobial class	Isolate number															
		PA84	PA123	PA148	PA183	PA208	PA212	PA220	PA232	PA241	PA263	PA420	PA447	PA457	PA498	PA508	PA527
Target alteration																	
* RmtF*	aminoglycosides									100%							
gyrA	fluoroquinolones	T83I	T83I	T83I	T83I	S80I, T83I	T83I	T83I		S80I	T83I		T83I	T83I	T83I	T83I	T83I
parC						S83I				S83I							
parE													A473V				
* PBP3*	β-lactams					D350N, S357N				D350N, S357N							
Target protection																	
* QnrB1*	fluoroquinolones					100%				100%							
* QnrS2*								100%									
Reduced permeability to antibiotic																	
* OmpK37*	β-lactams					M70I, M128I				M70I, M128I							

Shaded cells indicate presence of gene in the isolates. gyr: DNA gyrase; ParC: DNA topoisomerase IV subunit A; ParE: DNA topoisomerase 4 subunit B; Qnr: quinolone resistance pentapeptide repeat protein; RmtF: 16S rRNA methyltransferase. PBP; penicillin binding protein.

Amino acids: A; alanine, D; aspartic acid, I; isoleucine, M; methionine, N; asparagine, S; serine, T; threonine, V; valine. 100%; the gene is identical.

## Discussion

The present study describes MDR-*P. aeruginosa* bacteraemia over 3 years in Qatar. The proportion of MDR from all bloodstream isolates was 4.4%. This is small compared with reports from other regions such as Spain and Italy [2,14]. Not surprisingly, co-morbidities were highly prevalent in our patients and all had severe clinical presentations including septic shock [15]. However, our overall mortality was (5/16) at 30-days. Similarly, high mortality rates have been consistently reported with *P. aeruginosa* bacteraemia [16,17].

Antimicrobial resistance mutations can result in loss of fitness and reduced *P. aeruginosa* virulence [1]. However, from previous studies of invasive MDR-*P. aeruginosa* disease the production of potent virulence factors, such as exoU type III secretion system, have been linked to poor clinical outcomes in association with MDR-*P. aeruginosa* infections, but in the present study all cases of death express common virulence factor genes such as exoT and exoY with variable expressions of exoS and exoU which cannot be reliably linked to mortality (Table 3) [18]. Other important risk factors for mortality in patients with *P. aeruginosa* bacteraemia include multiple co-morbidity states, critical illness, shock, and older age, all of which were common in our patients [19,20].

Similar to the previous studies, colistin was the only agent with consistent *in-vitro* activity against MDR-*P. aeruginosa* bloodstream isolates included in the present study [21,22]. However, the clinical use of colistin is fairly problematic given its toxicity and the continuing uncertainty about its appropriate dosing [23]. The high prevalence of resistance to antipseudomonal β-lactam agents in this study is associated with the presence of class A ESBL (i.e.; *bla*_VEB-9_, *bla*_CTX-M-15_), MBL, and PDC enzymes. Vietnamese extended-spectrum β-lactamase (VEB) and Verona integron-encoded metallo-β-lactamase (VIM) enzymes are established in *P. aeruginosa* from our region [24]. *bla*_NDM_ and *bla*_IMP_, both of which are occasionally identified in MDR-*P. aeruginosa* from our region, were not detected in this study [24,25]. The majority of isolates were susceptible to aztreonam, which is not susceptible to the hydrolytic activity of MBLs or the narrow-spectrum OXA β-lactamases found in this study [26].

Ceftazidime/avibactam and ceftolozane/tazobactam were active against 8/16 MDR-*P. aeruginosa* isolates reported here. The study pre-dated their availability for clinical use in Qatar and none of the patients in this cohort was treated with these agents. Clinical data on the use of ceftazidime/avibactam and ceftolozane/tazobactam for the treatment of patients with infections caused by MDR-*P. aeruginosa* are mainly derived from retrospective studies [27,28]. Though the data are encouraging, their utility depends on the local epidemiology and prevailing *P. aeruginosa* resistance mechanisms [29]. Predictably, neither agent was active against VIM-producing MDR-*P. aeruginosa* isolates reported here (Tables 1 and 4).

All isolates included in this study were resistant to ciprofloxacin. Rates of fluoroquinolone resistance in MDR-*P. aeruginosa* are usually very high [30]. Fluoroquinolone resistance is predominantly mediated by well-characterized mutations in the *gyrA* (T83I), *parE* (A473V), and topoisomerase IV encoding genes, in addition to upregulation of efflux mechanisms [1,31]. Two types of transferable quinolone resistance genes, *Qnr* and *CrpP*, were also detected in this study. *Qnr*-encoding gene confers fluoroquinolone through target protection, while *CrpP*-mediates antibiotic modification through phosphorylation [32]. A variety of *Qnr*-encoding genes in *P. aeruginosa* have been described from multiple countries in the Arabian Peninsula and North Africa [33]. However, to our knowledge, the presence of *CrpP*-encoding gene in *P. aeruginosa* isolates from this region has not been previously reported.

*In-vitro* susceptibility of MDR-*P. aeruginosa* included in this study to aminoglycosides ranged from 31.3% for gentamicin to 43.8% for amikacin. Aminoglycoside resistance is driven mainly through the production of a variety of well-established AME [1]. Considering the antimicrobial agents available for clinical use at the time of the study, five (25%) isolates were only susceptible to colistin and an aminoglycoside. However, the efficacy of monotherapy with either of these for BSIs is questionable; their combined use is associated with an increased risk of toxicity [23].

A wide variety of sequence types were represented in this study’s MDR-*P. aeruginosa* bloodstream isolates, including the high-risk ST233 and ST357 [34]. Other previously reported high-risk *P. aeruginosa* clones such as ST235, ST111, and ST175 were notably absent [1].

## Conclusions

MDR-*P. aeruginosa* BSIs are relatively uncommon in Qatar, representing less than 5% of *P. aeruginosa* blood isolates over three years. Typical risk factors for MDR infections were highly prevalent in the study population and overall clinical outcomes are consistent with those previously reported. Its multiple clinical limitations notwithstanding, colistin was the only agent with consistent antibacterial activity against the study isolates. Alternatives, such as newer β-lactam-β-lactamase inhibitor combinations and aminoglycosides, are active against half of the isolates or less.

## Ethical approval

This study was approved by the Research Ethics Committee (Protocol number IRGC-01-51-033) at Hamad Medical Corporation (HMC), Doha, Qatar, with a waiver for informed consent.

## Supplementary Material

Supplemental Material

## Data Availability

The datasets used and analyzed during the current study are available from the corresponding author on reasonable request.

## Abbreviations

AMEs: Aminoglycoside modifying enzymes
ANT: aminoglycoside nucleotidyltransferases
APH: aminoglycoside phosphotransferases
BSIs: Bloodstream infections
CLSI: Clinical Laboratory Standards Institute
ESBL: extended-spectrum β-lactamase
MBL: metallo-β-lactamase
MIC: minimum inhibitory concentration
MDR: multi-drug resistant
PDC: *Pseudomonas*-derived cephalosporinase
ST: sequence type
VEB: Vietnamese extended-spectrum β-lactamase
VIM: Verona integron-encoded metallo-β-lactamase
WGS: whole-genome sequencing
WHO: World Health Organisation.
