# Supplementary material for: Clinical outcomes, molecular epidemiology and resistance mechanisms of multidrug-resistant Pseudomonas aeruginosa isolated from bloodstream infections from Qatar
Source: Ann Med. 2021 Dec 9;53(1):2345–53. doi: 10.1080/07853890.2021.2012588 (PMC8667892; doi:10.1080/07853890.2021.2012588)
Supplement: Supplemental Material [file IANN_A_2012588_SM7013.docx]

**Additional file 1**

**Clinical outcomes, molecular epidemiology and resistance mechanisms of multidrug-resistant *Pseudomonas aeruginosa*** **isolated from bloodstream infections from Qatar**

Mazen A. Sid Ahmed^a,b^*****, Jemal M. Hamid^a^, Ahmed A. Husain^c,d^, Hamad A. Hadi^c,d^, Sini Skariah^e^, Ali A. Sultan^e^, Emad B. Ibrahim^a,f^, Abdul Latif Al Khal^c,d^, Bo Soderquist^g^, Jana Jass^b^, Ali S. Omrani^c,d^

^a^Division of Microbiology, Department of Pathology and Laboratory Medicine, Hamad Medical Corporation, Doha, Qatar

^b^The Life Science Centre, School of Science and Technology, Örebro University, Örebro, Sweden

^c^Division of Infectious Diseases, Department of Medicine, Hamad Medical Corporation, Doha, Qatar

^d^Communicable Diseases Center, Hamad Medical Corporation, Doha, Qatar

^e^Department of Microbiology and Immunology, Weill Cornell Medicine-Qatar, Doha, Qatar

^f^Biomedical Research Centre, Qatar University, Doha, Qatar

^g^School of Medical Sciences, Faculty of Medicine and Health, Orebro University, Orebro, Sweden

**Table S1.** Genotypic profile of efflux pump complexes and their regulators of MDR *P. aeruginosa* isolated from blood in Hamad Medical corporation, Qatar.

| **Resistance genes** | **Isolate number** | | | | | | | | | | | | | | | |
| --- | --- | --- | --- | --- | --- | --- | --- | --- | --- | --- | --- | --- | --- | --- | --- | --- |
|  | **PA84** | **PA123** | **PA148** | **PA183** | **PA208** | **PA212** | **PA220** | **PA232** | **PA241** | **PA263** | **PA420** | **PA447** | **PA457** | **PA498** | **PA508** | **PA527** |
| **Efflux pump complexes** | **Gene presence** | | | | | | | | | | | | | | | |
| acrD | **+** | **−** | **−** | **−** | **−** | **−** | **−** | **−** | **−** | **−** | **−** | **−** | **−** | **−** | **−** | **−** |
| adeF | **+** | **+** | **−** | **+** | **+** | **+** | **+** | **−** | **+** | **−** | **−** | **−** | **+** | **−** | **−** | **−** |
| AxyXY-OprZ | **−** | **−** | **−** | **−** | **−** | **−** | **+** | **−** | **−** | **−** | **−** | **−** | **−** | **−** | **−** | **−** |
| MexAB-OprM | **+** | **+** | **+** | **+** | **+** | **+** | **+** | **+** | **+** | **+** | **+** | **+** | **+** | **+** | **+** | **+** |
| MexCD-OprJ | **+** | **+** | **+** | **+** | **+** | **+** | **+** | **+** | **+** | **+** | **+** | **+** | **+** | **+** | **+** | **−** |
| MexEF-OprN | **−** | **+** | **+** | **+** | **+** | **+** | **+** | **+** | **+** | **+** | **+** | **+** | **+** | **+** | **+** | **+** |
| MexGHI-OpmD | **−** | **+** | **+** | **+** | **+** | **+** | **+** | **+** | **+** | **+** | **+** | **+** | **+** | **+** | **+** | **−** |
| MexJK-OpmH | **+** | **+** | **+** | **+** | **+** | **+** | **+** | **+** | **+** | **+** | **+** | **+** | **+** | **+** | **+** | **+** |
| MexMN-OprM | **+** | **+** | **+** | **+** | **+** | **+** | **+** | **+** | **+** | **+** | **+** | **+** | **−** | **+** | **+** | **−** |
| MexPQ-OpmE | **+** | **+** | **+** | **+** | **+** | **+** | **+** | **+** | **+** | **+** | **+** | **+** | **+** | **+** | **+** | **−** |
| MexVW-OprM | **−** | **+** | **+** | **+** | **+** | **+** | **+** | **+** | **+** | **+** | **+** | **+** | **+** | **+** | **+** | **−** |
| mexY | **−** | **+** | **+** | **+** | **+** | **+** | **+** | **+** | **−** | **+** | **+** | **+** | **−** | **+** | **+** | **−** |
| mdtH | **+** | **−** | **−** | **−** | **−** | **−** | **−** | **−** | **−** | **−** | **−** | **−** | **−** | **−** | **−** | **−** |
| msbA | **−** | **−** | **−** | **−** | **+** | **−** | **−** | **−** | **+** | **−** | **−** | **−** | **−** | **−** | **−** | **−** |
| MuxABC-OpmB | **+** | **+** | **+** | **+** | **+** | **+** | **+** | **+** | **+** | **+** | **+** | **+** | **+** | **+** | **+** | **−** |
| oqxA | **−** | **−** | **−** | **−** | **+** | **−** | **−** | **−** | **+** | **−** | **−** | **−** | **−** | **−** | **−** | **−** |
| PmpM | **−** | **+** | **+** | **+** | **+** | **+** | **+** | **+** | **+** | **+** | **+** | **+** | **+** | **+** | **+** | **+** |
| tet(D) | **−** | **−** | **+** | **−** | **+** | **+** | **+** | **−** | **−** | **−** | **−** | **−** | **−** | **−** | **+** | **−** |
| TriABC-OpmH | **+** | **+** | **+** | **+** | **+** | **+** | **+** | **+** | **+** | **+** | **+** | **+** | **+** | **+** | **+** | **+** |
| **Efflux pump regulators** | | | | | | | | | | | | | | | | |
| AcrS | **+** | **−** | **−** | **−** | **−** | **−** | **−** | **−** | **−** | **−** | **−** | **−** | **−** | **−** | **−** | **−** |
| ArmR | **−** | **−** | **−** | **−** | **−** | **−** | **+** | **−** | **−** | **+** | **−** | **−** | **−** | **−** | **−** | **−** |
| baeR | **−** | **−** | **−** | **−** | **+** | **−** | **−** | **−** | **−** | **−** | **−** | **−** | **−** | **−** | **−** | **−** |
| baeS | **−** | **+** | **−** | **−** | **−** | **−** | **−** | **−** | **−** | **−** | **+** | **−** | **−** | **−** | **−** | **−** |
| bcr-1 | **−** | **+** | **+** | **+** | **+** | **+** | **+** | **+** | **+** | **+** | **+** | **+** | **+** | **+** | **+** | **−** |
| cmlA5 | **−** | **−** | **+** | **−** | **−** | **−** | **−** | **−** | **−** | **−** | **−** | **−** | **−** | **−** | **+** | **−** |
| CRP | **−** | **−** | **−** | **−** | **+** | **−** | **−** | **−** | **+** | **−** | **−** | **−** | **−** | **−** | **−** | **−** |
| emrB | **−** | **−** | **−** | **−** | **+** | **−** | **−** | **−** | **+** | **−** | **−** | **−** | **−** | **−** | **−** | **−** |
| emrK | **−** | **+** | **−** | **−** | **−** | **−** | **−** | **−** | **−** | **−** | **−** | **−** | **−** | **−** | **−** | **−** |
| emrR | **−** | **−** | **−** | **−** | **+** | **−** | **−** | **−** | **−** | **−** | **−** | **−** | **−** | **−** | **−** | **−** |
| emrY | **−** | **+** | **−** | **−** | **−** | **−** | **−** | **−** | **−** | **−** | **−** | **−** | **−** | **−** | **−** | **−** |
| *E. coli* marR mutant conferring antibiotic resistance | **−** | **−** | **−** | **−** | **+** | **−** | **−** | **−** | **+** | **−** | **−** | **−** | **−** | **−** | **−** | **−** |
| *E. coli* soxR with mutation conferring antibiotic resistance | **−** | **+** | **−** | **−** | **−** | **−** | **−** | **−** | **−** | **−** | **−** | **−** | **−** | **−** | **−** | **−** |
| evgS | **+** | **+** | **−** | **−** | **−** | **−** | **−** | **−** | **−** | **−** | **−** | **−** | **−** | **−** | **−** | **−** |
| floR | **−** | **−** | **−** | **−** | **+** | **+** | **−** | **−** | **−** | **−** | **−** | **−** | **−** | **−** | **−** | **−** |
| H-NS | **−** | **−** | **−** | **−** | **−** | **−** | **−** | **−** | **+** | **−** | **−** | **−** | **−** | **−** | **−** | **−** |
| mdtB | **−** | **+** | **−** | **−** | **−** | **−** | **−** | **−** | **−** | **−** | **−** | **−** | **−** | **−** | **−** | **−** |
| MexL, MexR, MexS, MexT, MexZ, nalC, nalD, *P. aeruginosa* CpxR, *P. aeruginosa* emrE, *P. aeruginosa* soxR | ***** | **+** | **+** | **+** | **+** | **+** | **+** | **+** | **+** | **+** | **+** | **+** | ***** | **+** | **+** | **+** |
| Type A NfxB | **−** | **−** | **+** | **+** | **+** | **+** | **+** | **+** | **+** | **+** | **+** | **+** | **+** | **+** | **+** | **−** |
| Type B NfxB | **−** | **+** | **−** | **−** | **−** | **−** | **−** | **−** | **−** | **−** | **−** | **−** | **−** | **−** | **−** | **−** |

^*^Deletion of MexR, MexZ, nalC
